# Prospective Association of Morning Salivary Cortisol with Depressive Symptoms in Mid-Life: A Life-Course Study 

**DOI:** 10.1371/journal.pone.0077603

**Published:** 2013-11-12

**Authors:** Marie-Claude Geoffroy, Clyde Hertzman, Leah Li, Chris Power

**Affiliations:** 1 Medical Research Council Center of Epidemiology for Child Health, Centre for Paediatric Epidemiology and Biostatistics, University College London Institute of Child Health, University College London, United Kingdom; 2 McGill Group for Suicide Studies, Douglas Mental Health University Institute, McGill University, Montreal, Canada; 3 School of Population and Public Health, Human Early Learning Partnership, University of British Columbia, Vancouver, Canada; National Taiwan University, Taiwan

## Abstract

**Objective:**

Associations of cortisol and depression vary at different life-stages, yet population-based, prospective studies are scarce. We aimed to assess associations of morning cortisol with depressive symptoms in mid-life taking account of lifetime psychological health.

**Methods:**

Participants were 5,403 men and women from the 1958 British Birth Cohort whose salivary cortisol was assessed at 45y (45min after waking (T_1_) and 3h later (T_2_)) and who completed the 5-item Mental-Health Index (MHI-5) about depressive symptoms at age 50y. Lifetime psychological health was identified from child and adult measures.

**Results:**

For women, higher T_2_ cortisol at 45y predicted depression (MHI-5 scores ≤52) at 50y (odds ratio [OR]=1.17; 95% confidence intervals [CI] 1.05,1.30 per standard deviation increase in T_2_ cortisol), attenuating when adjusted for current (45y) and previous (7-42y) psychological health (OR=1.11; 95% CI 0.98, 1.24). Similarly, an association in women of flatter cortisol delta (T_2_-T_1_) with depressive symptoms at 50y weakened after adjustment for current (45y) and previous (7-42y) psychological health. For men, lower T_2_ cortisol at 45y predicted greater depressive symptoms at 50y and the association strengthened when adjusted for lifetime psychological health. Likewise, lower cortisol AUC predicted higher risk of depression for men after adjusting for prior psychological health (OR=0.85; CI 0.72, 1.00). Associations were largely unaltered by control for covariates.

**Conclusions:**

In women, higher cortisol in late morning at 45y is prospectively associated with depressive symptoms at 50y through a link with lifetime psychological health. In men, lower cortisol predicts subsequent symptoms, independent of depressive history.

## Introduction

Major depression is the most common mental health illness[1] and a leading contributor to the global burden of disease[2]. Hypo- or hyper-activation of the hypothalamic-pituitary-adrenal (HPA) axis, with cortisol as the primary hormonal component, is frequently observed amongst depressed individuals[3]. It is biologically plausible that atypical secretion of cortisol may confer risk for depression as glucocorticoid receptors are widely distributed in brain areas implicated in emotion regulation, notably hippocampus and amygdala[4]. While HPA activity or cortisol secretion are suspected to influence the onset of depression, prospective studies are needed to verify this assumption. Of particular relevance for the putative role of cortisol on depression at a specific stage of life, is the growing evidence for an evolving relationship between cortisol and depression over the life-course[5]. In longitudinal studies of children and adolescents, hyperactivity of the HPA (as indicated by higher cortisol awakening response (CAR) and higher morning cortisol) has been shown to predict depression onset and recurrence[6–13]. However, findings from the few longitudinal studies in adulthood are less consistent, with studies showing that both lower and higher cortisol levels predict depression outcome[14–18]. For example, among 55 outpatients remitted from depression, lower morning cortisol predicted faster relapse of depression over a 5.5y period [17]. Whilst, in 116 initially not-depressed women (but at high risk for mental health difficulties) aged 23-58y, elevated morning cortisol at 8.00h was found to be associated with onset of major depressive episode in a 1y period[14] and, higher levels of cortisol after dexamethasone-CRH test predicted relapse in 74 inpatients remitted from depression[16]. The heterogeneity of adult studies may reflect the evolving relationship between cortisol and depression with age. If socio-emotional and HPA-axis development are co-founded early in life and then continue to co-evolve, earlier associations should be taken into account in investigations of cortisol and depression at later life stages.

 In contrast to child and adolescent populations, most depressed adults have already experienced more than one depressive episode[19]. The literature suggests that exposure to persistent psychopathology could alter the functioning of the HPA axis. Time elapsed since the onset of depression appears to be important in understanding the association between cortisol (hypo or hyper-secretion) and depression[20]. A few studies[21], including one based on the 1958 British Birth Cohort, have suggested that cortisol tends to be high around the time of depression onset, but lowered when depression persists for a long time[22]. Therefore, one challenge in investigating the longitudinal association between cortisol and depression, especially in adulthood, is to take account of previous life-course influences on cortisol including depressive symptoms.

In the present study, we examine whether salivary cortisol at 45y is associated with depressive symptoms at 50y in a general population sample. Because the association of cortisol with depression may develop over the life-course; we aim to investigate whether salivary cortisol is related to subsequent depression in mid-adulthood through a link with lifetime emotional status or whether cortisol has a separate contribution. 

## Materials and Methods

### Participants

The 1958 cohort includes all children born in England, Scotland, and Wales in 1 week in March 1958. About 17,000 live births were followed-up at ages 7, 11, 16, 23, 33, 42, 45 and 50y[23]. The 45y contact was a clinical examination undertaken by a trained research nurse; of 11,971 individuals invited, 9,377 participated. Written informed consent was obtained from all participants, whilst ethical approval for saliva collection and clinical examination at 45y was given by the South East Multi-Centre Research Ethics Committee.

### Salivary cortisol at 45y

Participants were asked to collect two saliva samples on the next convenient day after clinical examination, the first 45min after awaking before breakfast (time 1 or T_1_) and the second 3h later on the same day before lunch (time 2 or T_2_). Participants received a Home Saliva Collection Kit (including two numbered and color-coded salivette tubes, one pre-addressed envelope and instructions). They were instructed to collect saliva by chewing on a swab until it was soaked and to record the exact time of sample collection. Participants were asked to refrain from brushing or flossing their teeth and eating or drinking 15min before saliva collection and to store the sample at room temperature until posting to the laboratory. Salivary cortisol is stable at room temperature for up to 30 days, but samples were frozen after reaching the laboratory to reduce microbial growth. Cortisol levels were measured at the University of Dresden with a commercial chemiluminescence immunoassay kit (IBL International, Hamburg, Germany). The lower sensitivity of this assay is 0.44 nmol/L, with intraassay and interassay precision of <10% for a wide range of cortisol concentrations. High cortisol levels (>50 nmol/l) were rerun in a second assay for confirmation. 

#### Cortisol indicators

Extreme cortisol outliers were truncated at 2 nmol/l for <2 nmol/l (*n*=24 at T_1_, *n*=122 at T_2_) and at 100 nmol/l for >100 nmol/l (*n*=22 at T_1_, *n*=20 at T_2_) in order that extreme values did not exert a disproportionate influence on analyses. Not all samples were collected at the specified times, leading to variation around the target time for T_1_ [mean (S.D.)=49 (15)min] and T_2_ [mean (S.D.)=3h 5min (27min)]. Cortisol level was influenced by both the time of awakening and time since awaking, and to take account of this variability T_1_ value was centered at 08:08h (45min after a mean waking time of 07:22h) and T_2_ at 11:08h (3h 45min after mean awakening time) based on predictions from linear regression models at these time points[24]. Cortisol values had a skewed distribution and therefore were transformed using Log 10. As in prior publications with this cohort[22,24–27], we analysed several cortisol measures derived from transformed and centred values: first, T_1_ and T_2_ cortisol levels; second, cortisol T_2_ to T_1_ delta calculated as the difference between T_2_ and T_1_ cortisol levels divided by 3h; and third, area-under-the-curve (AUC), derived as the sum of T_1_ and T_2_ cortisol multiplied by 3h and divided by 2 (thus, AUC represents the 3h average of T_1_ and T_2_ values, allowing for variation in collection times, used here to indicate total 3h exposure). These measures were further converted into z-scores. Pearson correlation between T_1_ and T_2_ cortisol was 0.28, delta correlated at -0.79 with T_1_ and at 0.38 with T_2_, and AUC correlated at 0.88 with T_1_ and at 0.70 with T_2_, all p<.001.

#### Cortisol testing conditions

Information was collected on whether participants were awake during the previous night between 24:00 and 06:00, worked at night between 24:00 and 6:00, had dental work within the last 3 days or had cuts or other damage inside their mouth that may bleed, current medication affecting cortisol, and the day of the week of cortisol collection (weekend/weekday). Information on smoking habits (never/ex-/current smoker) was gathered at 42y. Day of week, current medication, smoking habits and night working were associated with T_1_ or T_2_ cortisol, and were controlled in the analyses. 

### Depressive symptoms outcome at 50y

Participants completed the Mental Health Inventory (MHI-5); a validated and widely used screening questionnaire for depression[28]. The 5 self-report items scored on a 6-point scale ranging from “all” to “none” of the time include: How much of the time during the last month have you: (1) been a very nervous person?; (2) felt calm and peaceful?; (3) felt downhearted and low?; (4) been a happy person; (5) felt so down in the dumps that nothing cheers you up? The scale was scored 0-100, with lower scores indicating more severe symptoms. The MHI-5 has been shown to have high sensitivity and specificity in detecting clinical depression[29]. 

#### Categorical measure

As in previous studies, MHI-5 scores ≤52 were identified as elevated depressive symptoms (hereafter referred to as ‘depression’) and scores>52 as not depressed [30,31]. 

#### Continuous measure

To facilitate interpretation, the original MHI-5 scale was reversed so that those with the most severe symptoms had higher scores. 

### Depressive symptoms (45y)

Symptoms in the past week (persistent sadness/low mood and/or marked loss of interest/pleasure for ≥4days/week and/or ≥3h/day and/or no reactivity to pleasurable stimuli) were established with the widely used Clinical Interview Schedule (CIS-R), administered by a trained nurse visiting during the clinical examination[32]. Scores range from 0-4 symptoms. 

### Previous adulthood depressive symptoms (23-42y)

A summary score of adulthood symptoms was calculated as the mean of psychological malaise scores reported by participants at ages 23, 33 and 42y. The Psychological Malaise[33] comprised 15 items covering major components of depression and anxiety, including depressed mood, sleep disturbance, fatigue, irritability, excessive worries, and phobias scored as 0 (no) to 1 (yes). Individual scores at 23, 33, and 42y were converted into z-scores. A summary score was calculated for participants with a malaise score at ≥1 age (78.8% had data at all three ages).

### Childhood (7-11y) and adolescent (16y) behaviour

A summary childhood and adolescent behaviour problems score was calculated as the mean of internalizing and externalizing behaviours scores as assessed by teachers using the Bristol Social Adjustment Guide (BSAG) at 7 and 11y[34] and by the Rutter Behaviour Scale at 16y[35]. The BSAG consists of 146 behaviour-items assessing 12 syndromes, grouped into internalizing (items such as miserable, fearful) and externalizing (e.g. resentful/aggressive, bullies) scores, participants had a score of 1 if an item applied and 0 if it did not apply. The Rutter Scale consists of 26 behaviour-items, five of which were used to derive an internalizing score (e.g. worries, solitary, miserable) and nine for an externalizing score (e.g. destructive, irritable, disobedience, fighting); participants had a score of 2 if an item applied ‘definitely’, 1 for ‘somewhat’ and 0 if it did not apply. Individual scores at 7, 11, and 16y were converted into z-scores. A summary score was calculated for participants with a behaviour score at ≥1 age (64.7% had data at all three ages). 

### Covariates

Several covariates were examined, including socio-economic position (SEP) in childhood and in adulthood, birth-weight, body mass index (BMI; at 45y), cholesterol (45y), hypertension (45y), post-menopausal status (45y), limiting illness (42y), heavy drinking (45y), physical activity (42y), childhood and adulthood adversity. We controlled for those associated with both cortisol exposure (T_1_ or T_2_) and depressive symptoms (categorical measure): childhood SEP (based on father’s social class at birth or 7y if missing: I/II, IIINM, IIIM, IV/V) and adulthood SEP (from participant’s class at 42y or 23-33y if missing: I/II, IIINM, IIIM, IV/V), BMI (measured weight in kilograms divided by squared height in meters), limiting long-term illness (no/yes) and childhood and adulthood adversity. For childhood adversity, participants answered confidential questions at 45y, about their childhood to age 16y, from the Path Through Life Project[36] including abuse by a parent (physical, sexual, psychological), witnessing intimate partner violence and neglect by a parent (neglect, lack of father or mother affection). A categorical score (0, 1, 2, or ≥3) was created by counting the reports of adversities[24]. For adult adversity at 45y, participants reported whether they had experienced (yes/no) during the preceding six months any of the following life events: serious illness/injury/assault to you or close friend/relative; death of a family member or relative/friend; separation or divorce; crisis or serious disappointment at work; sacked from work, thought of losing job soon; unemployed/seeking work for >1 month; major financial crisis; problem with police and court appearance; something valued was lost or stolen. A categorical score (0, 1, 2, or ≥3) was created by summing stressful life events experienced. 

### STATISTICAL ANALYSES

The main analyses examined associations between morning cortisol indicators (T_1_, T_2_, delta T_2_-T_1_, AUC) at 45y and depressive symptoms at 50y, using logistic regression for the categorical (MHI-5 score ≤52 versus >52) outcome and linear regression for the continuous outcome (reversed-score with higher values indicating more severe symptoms and transformed to z-score to ease interpretation) using robust standard errors to account for the skewed distribution of MHI-5. Models for depressive symptoms (categorical and continuous) were adjusted for cortisol testing conditions (model 1); 45y depressive symptoms (model 2), previous adulthood (23-42y) depressive symptoms and childhood/adolescent (7-16y) behavioural problems (model 3); and other covariates (model 4). We tested whether associations between cortisol and depressive symptoms (categorical outcome) differed for men and women using an interaction term for sex. 

To illustrate associations between cortisol levels and the course of depressive symptoms, 45y to 50y, we created a 4-category variable by cross-classifying presence or absence of symptoms at 45y (with a cut-off of ≥2 symptoms on the CIS-R[32]) and 50y (MHI-5 score ≤52). “Not depressed”, the reference category, included participants with low symptoms level at 45 and 50y (i.e. CIS-R score <2 and MHI-5 score >52); “remitted” included those with a CIS-R score ≥2 and MIH-5 score >52; “incident” included a CIS-R score <2 and MHI-5 score ≤52; “persistent” included individuals with elevated symptoms at both 45 (CIS-R score ≥2) and 50y (MHI-5 score ≤52). We estimated the geometric mean for T_1_ and T_2_ cortisol adjusted for all covariates using analyses of covariance (ANCOVA) with type III sum of squares. Statistical differences in adjusted geometric means for each category (remitted/incident/persistent symptoms) versus “not depressed” were estimated for T_1_ and T_2_ cortisol and tested using Bonferonni correction. 

Eligible participants for this study included those with MHI-5 data at 50y (*n*=8762); information on cortisol was available for 5403 of these participants (62% of 8762), of whom 826 had incomplete information on covariates. To assess whether our findings were affected by sample attrition between ages 45y and 50y or missing data, we conducted three separate analyses. First, we used multiple imputation by chained equations methods to impute missing values on covariates for 826 participants (but not on exposure or outcome). We created 10 datasets, and conducted regression analyses that combined results from these datasets. Second, we applied inverse probability weighting relative to surviving cohort members resident in the UK by 31^st^ May 2009. The probability of being in the study sample was estimated from logistic regressions using factors associated with sample attrition including sex, SEP at birth, math scores and internalizing and externalizing behaviors at 7y[37]. Third, we repeated analyses using the sample with complete data. The pattern of results was similar for complete data (*n*=4577), weighted (*n*=4504) and imputed analyses (*n*=5403); the latter are presented here to maximize number of participants. 

## Results

**Figure 1:**
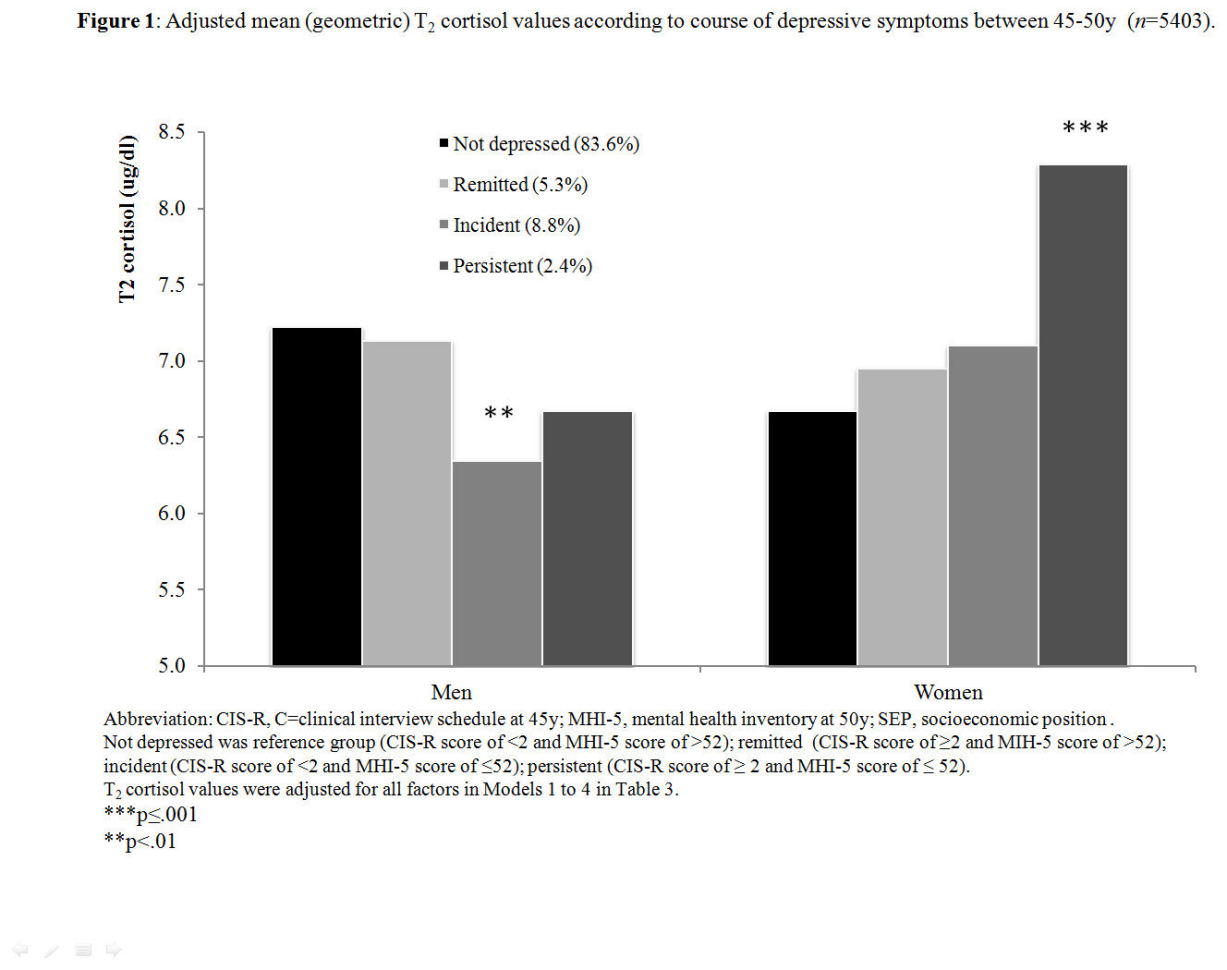
Adjusted mean (geometric) T_2_ cortisol values according to course of depressive symptoms between 45-50y (*n*=5403).

Table **1** presents a description of the study sample on key variables for men and women separately. Table **2** shows that T_1_ cortisol was lower and T_2_ cortisol elevated, although not always significantly, for the depressed group at different life-stages compared to others. Table **3** shows results from logistic and linear regressions assessing prospective associations of cortisol at 45y with depressive symptoms (categorical and continuous) at 50y. Some interaction terms were found (p<0.05, Table 3), hence analyses were conducted separately for men and women. For women, with adjustment for testing conditions only, elevated T_2_ cortisol was associated with depressive symptoms (odds ratio [OR]=1.17; 95% confidence intervals [CI] 1.05, 1.30 for categorical outcome; β=0.07, *p*=.019 for continuous outcome). Adjustment for current (45y) and previous psychological health (7-42y) weakened these associations (OR=1.11; CI 0.98, 1.24, *p*=.091 for categorical outcome; β=0.03, *p*=.227 for continuous outcome). Similarly, an association of flatter cortisol delta with depressive symptoms at 50y, weakened after adjustment for 45y symptoms and was then abolished with further adjustment for earlier psychological health. For men, T_2_ cortisol was not associated with depressive symptoms at 50y in models adjusted only for testing conditions, but the association strengthened after taking account of lifetime psychological health (OR=0.85; CI 0.71, 1.02, *p*=.075 for categorical outcome; β=-0.04, *p*=.008 for continuous outcome) and remained after controlling for covariates. A similar pattern of association was detected for cortisol AUC, with highest risk of subsequent depressive symptoms for men with lower AUC (OR=0.85; CI 0.72, 1.00, *p*=.050 for categorical outcome; β=-0.02, *p*=.095 for continuous outcome) evident after adjusting for prior psychological health. Figure **1** presents fully adjusted T_2_ cortisol values for depressive symptoms groups (remitted/incident/persistent *vs* not depressed). In men, T_2_ cortisol levels were lower for incident symptoms versus not depressed (adjusted geometric mean=6.34 (95% CI 5.80,6.93) *vs* 7.22 (7.03,7.41), *p*=.007) but not for remitted or persistent symptoms. In women, T_2_ cortisol levels were higher for persistent depressive symptoms versus not depressed (adjusted geometric mean=8.29 (95% CI 7.32,9.39) *vs* 6.67 (6.53,6.82), *p*=.001) but not for remitted or incident symptoms. Finally, there was no prospective association of T_1_ cortisol with depressive symptoms for men or women (Table **3**) and no difference in adjusted T_1_ cortisol between 45-50y symptom groups (remitted/incident/persistent *vs* not depressed; data not shown).

**Table 1:** Description of the 1958 British Birth Cohort on key characteristics (n=5403).

	Men	Women
	(*n*=2576)	(*n*=2827)
**Salivary cortisol at 45y**, **mean** (**s.d.**)^a^		
T_1_ cortisol (nmols/l) (T_1_) (2 to 100)	20.86 (11.51)	21.58 (11.39)
T_2_ cortisol (nmols/l) (T_2_) (2 to 100)^c^	8.91 (8.22)	7.97 (6.72)
Delta from T_2_ to T_1_ (-31 to 29 nmols/l)	-3.99 (3.97)	-4.54 (4.04)
AUC (range 6 to 300 nmols/l)	44.66 (24.10)	44.33 (21.34)
**Depressive symptoms at 50y**		
Depressed (MHI-5≤52) % (*n*)	9.7 (249)	12.5 (354)
Mean (s.d) reversed scores (0 to 100)	22.44 (16.73)	24.90 (17.56)
**Depressive symptoms at 45y**		
Depressed (CIS-R≥2) % (*n*)	7.1 (182)	8.2 (232)
Mean (s.d) scores (range 0 to 4)	0.25 (0.71)	0.29 (0.74)
**Previous psychological health covariates, mean (s.d.)**		
Adulthood depressive symptoms (23-42y) (-0.93 to 4.46)	-0.24 (0.68)	0.06 (0.83)
Childhood behavioural problems (7-16y) (-1.78 to 2.92)	-0.05 (0.58)	-0.21 (0.49)
**Course of depressive symptoms between 45-50y, % (*n*)**		
Not depressed between 45-50y (CIS-R<2 and MHI-5>52)	85.4 (2199)	82.0 (2317)
Remitted (CIS-R≥2 and MHI-5>52)	5.0 (128)	5.5 (156)
Incident (CIS-R<2 and MHI-5≤52)	7.6 (195)	9.8 (218)
Persistent (CIS-R≥2 and MHI-5≤52)	2.1 (54)	2.7 (76)
**Other covariates, % (*n*)**		
**Father's SEP at birth**		
I & II	20.8 (537)	20.9 (592)
IIInm	11.1 (287)	10.3 (290)
IIIm	47.7 (1228)	48.0 (1358)
IV & V	20.3 (524)	20.8 (587)
**SEP in adulthood at 42y**		
I & II	47.8 (1231)	38.0 (1075)
IIInm	10.0 (258)	34.2 (968)
IIIm	30.8 (793)	7.1 (200)
IV & V	11.4 (294)	20.7 (584)
**Limiting illness at 42y**		
No	89.1 (2294)	88.4 (2500)
Yes	10.9 (282)	11.6 (327)
**Childhood adversity by 16y**		
None	82.6 (2129)	79.0 (2232)
1	10.6 (272)	10.5 (296)
2	3.6 (93)	5.1 (145)
≥ 3	3.2 (82)	5.4 (154)
**Adulthood adversity at 45y**		
None	51.4 (1323)	50.5 (1428)
1	26.3 (677)	28.1 (794)
2	12.7 (328)	12.5 (354)
≥ 3	9.6 (248)	8.9 (251)
BMI at 45y (kg/m^2^) (16.53 to 64.67), mean (s.d.)	27.81 (4.35)	26.81 (5.53)

Abbreviation: BMI, body mass index, CIS-R, clinical interview schedule-revised, MHI-5, mental health index- 5 items, SEP, socioeconomic position, S.D., standard deviation.

P-values for sex difference for T1=.022; for T2=<.001; for delta=<.001; for AUC=.600; for depressive symptoms at

50y= <.001; for depressive symptoms at 45y=.038; for adulthood depressive symptoms=<.001; for childhood behavioural problems=<.001; for course of depressive symptoms between 45-50y=<.001; for SEP at birth=.860; for SEP at 42y=<.001; for limiting illness at 42y=.230; for childhood adversity by 16y=<.001; for adulthood adversity at 45y=.330; for BMI at 45y=<.001.

**Table 2:** Mean (geometric) T1 and T2 cortisol values^**a**^ by depressive symptoms over the life-course (*n*=5403).

Depressive symptoms	Men (*n*=2576)				Women (*n*=2827)			
	**T_1_**		**T_2_**		**T_1_**		**T_2_**	
	Geometric mean							
**Subsequent symptoms (50y)**								
Depressed	18.25		7.18	†	19.04		6.67	***
Not depressed	17.37		6.72		17.97		7.46	
**Current symptoms (45y)**								
Depressed	18.14		7.12		18.91		6.70	**
Not depressed	18.50		7.33		18.88		7.52	
**Previous adult symptoms (23-42y)^#^**								
Depressed	18.18		7.07	**	19.15	**	6.71	†
Not depressed	17.90		8.24		17.41		7.12	
**Previous childhood symptoms (7-16y)^#^**								
Behaviour problems	18.17		7.08		18.96		6.76	
No behaviour problems	18.13		7.34		18.20		6.86	

*** *p*≤.001

** *p*<.01

† *p*<.10

# For the purposes of illustration, adult symptoms (23-42y) and childhood (7-16y) behavioural problems were categorized as below (not depressed) and above (depressed) the 10^th^ percentile.

**Table 3:** Association between cortisol at 45y and depressive symptoms at 50y (*n*=5403).

	Men (*n*=2576)			Women (*n*=2827)			
	Depressed *vs* not depressed	Symptoms^a^		Depressed *vs* not depressed		Symptoms^a^	
	OR (95% CI)	B (SE)		OR (95% CI)		B (SE)	
**Cortisol indicators at 45y (z-score)^a^**							
***T_1_ Cortisol***							
Model 1	0.89 (0.77,1.03)	0.00 (0.02)		0.94 (0.83,1.06)		-0.02 (0.02)	
Model 2	0.89 (0.77, 1.03)	0.00 (0.02)		0.93 (0.83,1.06)		-0.01 (0.02)	
Model 3	0.89 (0.76, 1.04)	0.00 (0.02)		0.98 (0.86, 1.11)		0.00 (0.02)	
Model 4	0.90 (0.77, 1.05)	-0.01 (0.02)		0.99 (0.88,1.13)		0.00 (0.02)	
***T_2_ Cortisol***							
Model 1	0.89 (0.76, 1.05)	-0.02 (0.02)		1.17 (1.05,1.30)	**	0.07 (0.03)	*
Model 2	0.88 (0.75, 1.03)	-0.03 (0.02)	†	1.14 (1.02,1.28)	*	0.05 (0.03)	*
Model 3	0.85 (0.71, 1.02)	-0.04 (0.02)	**	1.11 (0.98,1.24)	†	0.03 (0.02)	
Model 4	0.84 (0.71, 1.01)	-0.04 (0.02)	**	1.11 (0.98, 1.26)	†	0.03 (0.02)	
***Delta (T_2_-T1/3h)***							
Model 1	1.04 (0.90, 1.19)	-0.01 (0.02)		1.17 (1.05,1.31)		0.05 (0.02)	*
Model 2	1.02 (0.89, 1.18)	-0.02 (0.02)		1.15 (1.03, 1.29)		0.04 (0.02)	*
Model 3	1.01 (0.88, 1.16)	-0.02 (0.02)		1.09 (0.96, 1.22)		0.01 (0.02)	
Model 4	1.00 (0.86, 1.15)	-0.02 (0.02)		1.07 (0.95, 1.21)		0.01 (0.02)	
***AUC***							
Model 1	0.87 (0.75, 1.01)	-0.01 (0.02)		1.04 (0.92, 1.17)		0.02 (0.02)	
Model 2	0.86 (0.74, 1.01)	-0.02 (0.02)		1.02 (0.90, 1.15)		0.01 (0.02)	
Model 3	0.85 (0.72, 1.00)	-0.02 (0.01)		1.04 (0.92, 1.17)		0.01 (0.02)	
Model 4	0.85 (0.72, 1.00)	-0.02 (0.01)	†	1.05 (0.93, 1.19)		0.02 (0.02)	

Abbreviation: OR, odds ratio; CI, confidence intervals; SE, standard error.

a Reversed.

Model 1 adjusted for testing conditions: day (weekday/weekend) of cortisol collection, medication at 45y (no/yes), night work at 45y (no/yes), smoking at 42y (never/ex/current).

Model 2 adjusted for baseline depressive symptoms at 45y.

Model 3 adjusted for lifetime psychological health: summary score for externalizing/internalizing problems in childhood (7-16y), summary sore for psychological Malaise in adulthood (23-42y).

Model 4 adjusted for other covariates: lifetime social class (I&II/IIINM/IIIM/IV&IV),BMI at 45y, limiting illness at 42y (no/yes), childhood and adulthood life-adversity (0/1/2/≥3).

** *p*<.01

* *p*<.05

† *p*<.10

P-values for sex interaction for t1=.623; for t2=.006; for delta =.187; for AUC=.075 (calculated from model 1 with categorical depression outcome).

## Discussion

 Our main finding is that naturally occurring cortisol levels at 45y predicted subsequent depressive symptoms at 50y in both sexes, but with contributions of lifetime psychological health that differed in their impact for men and women. To the best of our knowledge, this is the first epidemiological study to show this. For women, higher late-morning cortisol (T_2_) at 45y predicted depressive symptoms at 50y. The association was abolished when taking account of current and past psychological health, because high T_2_ cortisol was associated with persistent depression at 50y. For men, lower late-morning cortisol (T_2_) at 45y predicted depressive symptoms at 50y. The association strengthened when allowance was made for earlier psychological health, because low T_2_ cortisol levels predicted incident depressive symptoms subsequently at 50y. 

### Methodological considerations

Data for the 1958 British Birth Cohort, including repeated assessments of depressive symptoms over the life-course, provided an opportunity to examine the longitudinal relationship from cortisol to depression in the general population. Previous longitudinal studies have relied on small or selected (e.g. high neuroticism trait) samples or psychiatric participants, limiting generalisation of findings. Furthermore, unlike previous studies relying predominantly on female populations, we were able to investigate the association between cortisol and depressive symptoms in men. The inclusion of several key covariates, such as childhood and adulthood adversities, is a further strength of the study. However, there are several study limitations. First, the MHI-5 is a validated screening questionnaire rather than a clinical depression diagnostic tool[28] and thus, identifies individuals with elevated symptoms; a mixture of subclinical, minor and major depression. However, emerging evidence suggests that the association of cortisol and depression is not confined to clinical depression, but extends to the broader range of symptoms[12,38,39]. Second, our 4-category variable for depressive symptoms 45 to 50y is imperfect, being based on different instruments (CIS-R and MHI-5) and change in symptoms between the 45 and 50y measurements may have been missed. Third, our cortisol indicators, based on two morning samples during 1 day, might not adequately capture an individual’s secretion level. Additional cortisol samples during the day or night would better indicate naturally occurring secretion patterns. It has been reported previously that afternoon cortisol or cortisol collected throughout the day produces a larger effect size with depression than morning cortisol measures[40]. Further, samples collected at earlier life-stages would inform understanding of the lifetime co-evolution of cortisol and psychological health. Fourth, although we controlled for numerous covariates, the possibility of unmeasured confounding remains. 

### Interpretation of findings

Our findings indicate that consideration of lifetime psychological health is essential to understand the relationship between cortisol level and subsequent depressive symptoms in mid-life. This is consistent with the suggestion that chronic stress or psychopathology may initially be associated with heightened cortisol response, but over time the HPA axis may be down-regulated and lead to attenuation of the cortisol response [20,21,41]. Such changes over the life-course can obscure the association of cortisol with depression, especially in adult life when individuals may have had a cumulative exposure to depression and/or when effects of cortisol may be over-shadowed by more dominant influences on depression, such as relationship or job loss. For women in our study, the abolition of an association of 45y cortisol with subsequent (50y) depressive symptoms that resulted from adjustment for symptom history is likely to reflect associations from earlier in life that persist through to ages 45-50y. However, our results showing that associations between cortisol at 45y and depressive symptoms at 50y were dependent on previous psychological health contradicts the findings of another study. Harris and al[14]. reported that elevated morning cortisol increased the risk of subsequent depression in adult women by 1.3 times independently of previous mental illness; their assessment of mental health history was retrospective and therefore less reliable than our prospective assessment. 

Associations of cortisol secretion with depressive symptoms in men have been largely neglected in the literature. While depression disproportionally affects women from the beginning to the end of reproductive life[42], men are more prone to depression with advancing age[43]; our findings suggest that a blunted morning cortisol pattern is a precursor to their mid-life depression. When we took account of lifetime psychological health trajectories, men with lower late morning cortisol levels at 45y were more likely to have onset of depressive symptoms by 50y. Associations between low basal cortisol and depression have been reported elsewhere. For example, in 152 (133 men) patients who had survived an acute coronary episode, blunted cortisol awakening response was associated with current depression, taking account of previous depression and other factors[44].

Another interesting finding of our study is that late-morning (T_2_), but not early morning (T_1_) cortisol was predictive of subsequent symptoms at 50y. Previously we showed, within this cohort, that elevated T_2_ cortisol (but not T_1_) at 45y predicted worse cognition at 50y . In childhood and adolescence, many studies reported that higher cortisol awakening response or early morning cortisol predicts depression onset and recurrence[6–9,11–13], whereas the very few prospective studies on cortisol and depression in adulthood show discrepant findings[14–17]. Whilst findings in the literature appear contradictory, they highlight the likelihood that associations of cortisol and mental health vary at different life-stages and by sex. Furthermore, there is no agreement in the literature on the specific component(s) of cortisol diurnal profile that indicates risk for later outcome and, as far as we are aware, the predictive role of late morning cortisol for outcomes has received little attention.

##  Conclusions

Naturally occurring cortisol was longitudinally associated with depressive symptoms in our large community sample of middle-aged adults, with earlier depressive symptoms contributing to this association. In women, associations between elevated cortisol and depressive symptoms appeared to develop before mid-life. This was the first study to find that, in men, blunted late-morning cortisol may precede the onset of depressive symptoms, 5y later. Future studies with repeated measurements of cortisol and depressive symptoms are needed to clarify further the temporality of the evolving HPA-axis and related mental health history. 
